# Surface reconstruction in gold nanowires

**DOI:** 10.1038/s41598-018-28145-y

**Published:** 2018-06-29

**Authors:** Yasuchika Suzuki, Tokushi Kizuka

**Affiliations:** Department of Materials Science, Faculty of Pure and Applied Sciences, University of Tsukuba, 1-1-1, Tennoudai, Tsukuba, Ibaraki 305-8573 Japan

## Abstract

Surface reconstructions are caused by structural stabilization resulting from the modulation of surface atomic positions. Studies on surface reconstruction have been conducted for substantially large surfaces, rather than at the size of reconstructed surface unit cells. Hence, well-known surface reconstruction manners may not be applicable for the surfaces of nanometer-sized isolated crystals, such as nanoclusters, nanowires and nanotubes. This is because they have high surface area-to-interior volume ratios exceeding several tens of percent, and their surface structures significantly affect the stabilization of their entire structures. In this study, we demonstrate the inherent surface reconstruction of gold nanowires via nanosecond-pulsed electromigration with the application of tensile stresses. The results lead to evolutions in basic studies relating to surface reconstruction and nanostructures and in applications of nanowires, for which stabilization is essential when they are used in extremely miniaturized integrated circuits for next-generation electronics.

## Introduction

The atomic configuration on crystal surfaces is modulated and unit structures different from ideal surfaces appear, as exemplified by the dimer-adatom-stacking fault model of silicon surface reconstruction^1^. Peculiar atomic configurations also emerge in nanometer-sized isolated structures, such as nanoclusters, nanowires (NWs) and nanotubes^1,2^: five-fold symmetric multiply twinned nanoclusters having a disclination^3,4^, nanotubes with helical structures^5^, gold (Au) NWs comprising a body-centered tetragonal structure^6^, amorphized pure metal nanocontacts (NCs)^7,8^, and atomic chains in which single atoms align on a line^9–12^. In addition to increased surface area-to-interior volume ratio effects^2,13^, such peculiar atomic configurations of surfaces and nanostructures lead to new non-bulky inherent properties, inspiring the creation of devices with unprecedented electrical, mechanical, optical, and magnetic functions^14–16^.

The specific nanostructures can be roughly classified into two types: those where the entire crystal structure changes such as NWs and nanotubes having helical structures and amorphized NCs^5,6,8,12^; and those where twins and specific crystal boundaries are introduced in nanostructures, such as multiply twinned nanoclusters^3,4^. In this study, we demonstrate a new type of nanostructure belonging to neither of these two types, i.e., the formation of NWs for which monatomic surface layers are stabilized via reconstruction by a stress-applied electromigration method.

## Results

### Fabrication of a Au NW via pulsed voltage application under tensile stress

**Figure 1:**
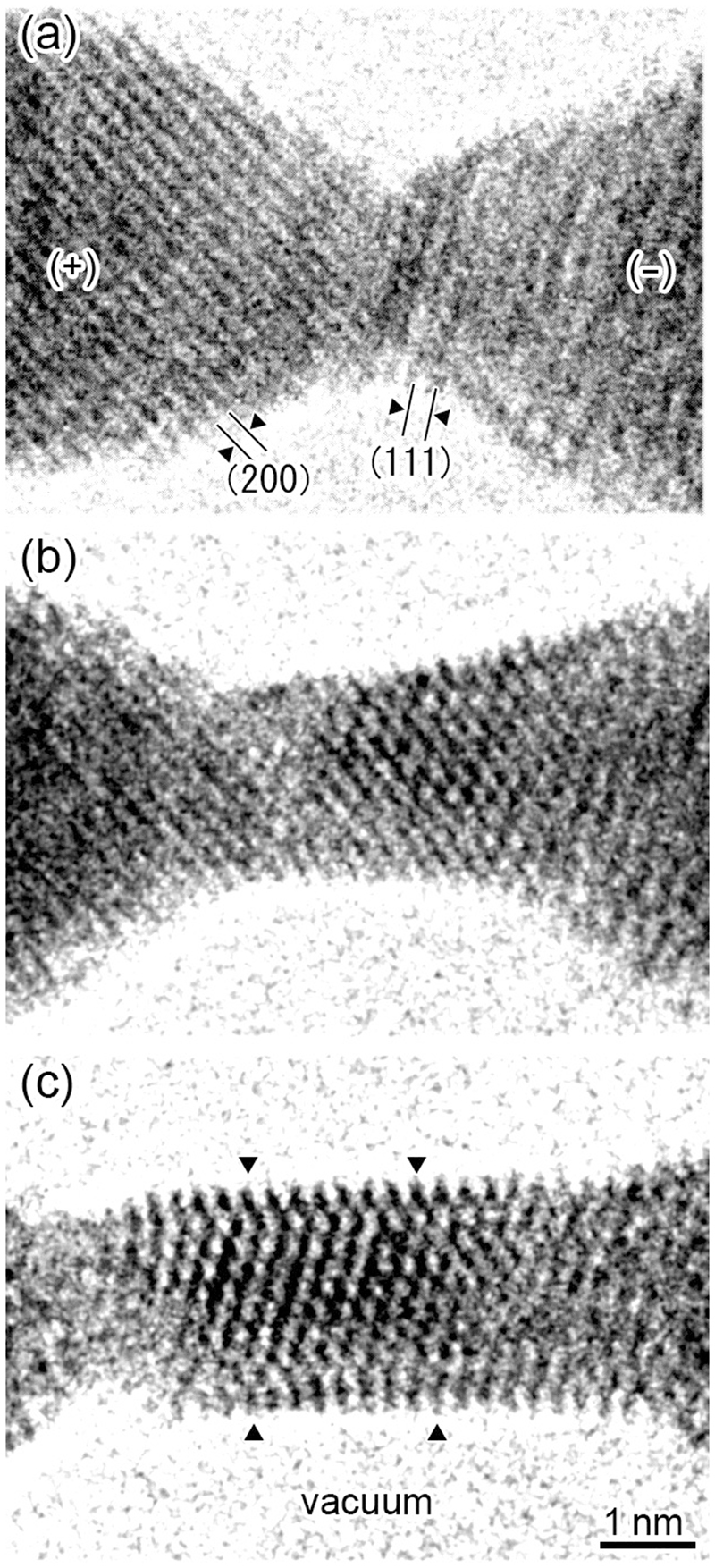
Time series of high-resolution images of the formation of a Au NW during pulsed voltage application under tensile stress. (**a**) A NC before the NW formation via pulsed voltage. The dark regions on the left and right sides of the images correspond to positively and negatively biased nanotips, indicated by (+) and (−), respectively. The NC is formed between the two nanotips. The (200) and (111) lattice fringes are observed on the left and right nanotips. The bright regions at the upper and lower sides of the NW correspond to the vacuum. (**b**) and (**c**) The states after 18 and after 38 pulses, respectively. The width and height of the applied pulse waves are 4 ns and 0.60 V, respectively. A NW of 2.3 nm in width and 6.4 nm in length is formed, as shown in (c).

Figure 1 shows a time series of high-resolution transmission electron microscope (TEM) images of the formation of a Au NW during pulsed voltage application under tensile stress (see Movie 1 for the observed dynamic image and Fig. S1 for the experimental setup in supplementary information). In Fig. 1a, a Au NC before the NW formation via pulse wave application is observed between the two nanotips in the left and right sides of the image. The (111) and (200) lattice fringes are observed on the right and left nanotips, which are negatively and positively biased, respectively. The bright regions at the upper and lower sides correspond to the vacuum. When pulsed voltages 4 ns in width and 0.60 V in height were applied to the NC shown in Fig. 1a, the positively biased side of the NC thinned, whereas the negatively biased side extended while maintaining its cross-sectional width. Thus, electromigration occurred from the positively biased side to the negatively biased side, resulting in growth of a NW via pulse application. The electromigration direction implies that the constriction region melts once during the pulse application^17^. The NW length increased to 3.1 nm after 18 pulses, as shown in Fig. 1b, and to 6.4 nm after 38 times pulsing, as shown in Fig. 1c. In this observation, we applied various pulsed waves to the NCs for the NW formation. When we selected pulsed voltages of longer than 4 ns in width and higher than 0.60 V in height, the NW formation became instable; the formation speed and resultant NWs width were liable to became inhomogeneous. Then, we selected the above condition, i.e., 4 ns in width (the minimum width in our system) and 0.60 V in height.

**Figure 2:**
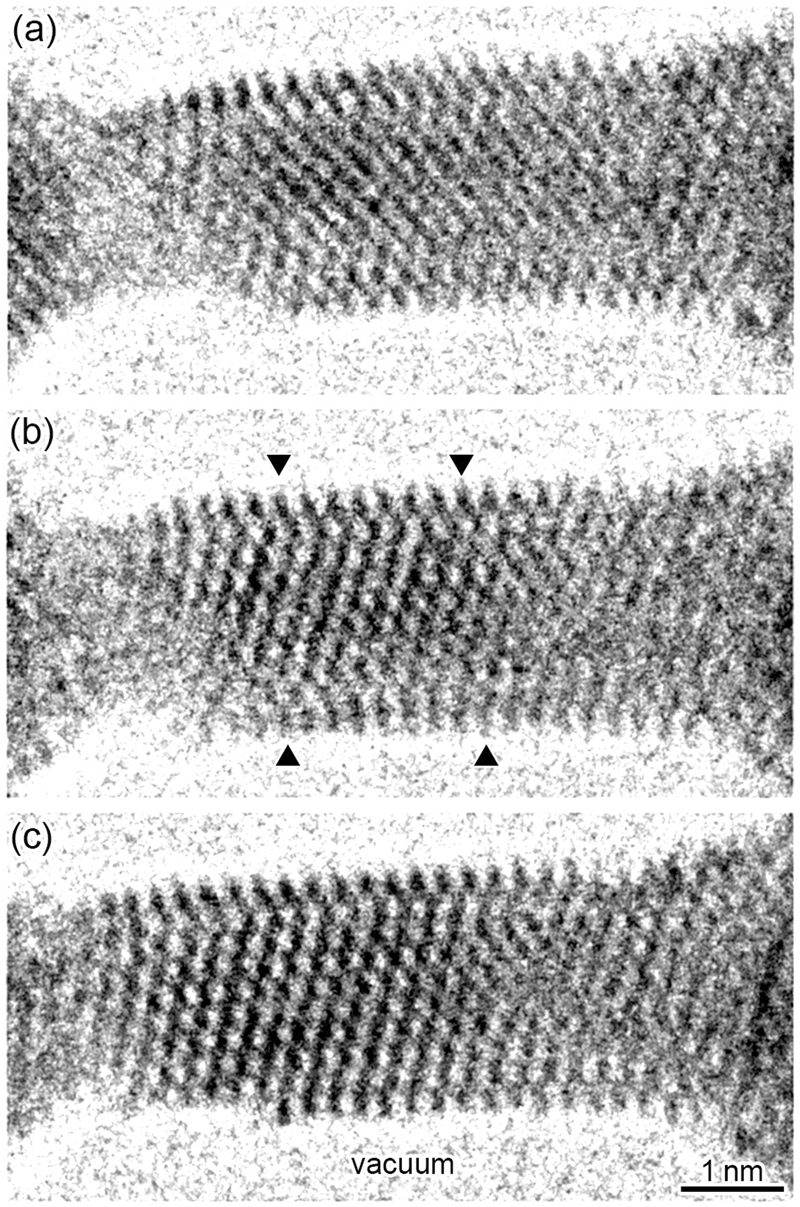
Time series of high-resolution images of the surface reconstruction and subsequent relaxation of the Au NW shown in Fig. 1c during one pulse. (**a**) High-resolution image before the application of the last pulse to the NW shown in Fig. 1c (i.e., after 37 pulses to the NC shown in Fig. 1a). (**b**) After a single pulse to a (an enlarged image of the NW shown in Fig. 1c). (**c**) Relaxation state 1 s after (b) in which the surface reconstruction at the bottom side surface disappears.

Figure 2 shows a time series of high-resolution TEM images of the surface reconstruction and subsequent relaxation of the Au NW shown in Fig. 1c during one pulse (see Movie 2 for the observed dynamic image in supplementary information). Fig. 2a shows the high-resolution image before the application of the last pulse to the NW shown in Fig. 1c, i.e., this structure corresponds to the state after the application of 37 pulses to the NC shown in Fig. 1a. When one of the single pulse waves was applied to the NW shown in Fig. 2a, atoms migrated, resulting in thinning of the positive side of the minimum cross-sectional region and thickening of the negative side of the NW. Note that the atomic configuration on the monatomic surface layer on the top side surface and two surface layers on the bottom side surface became disordered, i.e., surface reconstruction occurred. With the addition of a single pulse wave, the NW was elongated in the [211] growth direction, and the spacing of the (211) lattice planes was extended by 7% in comparison with that of the stress-free state, i.e., a tensile strain of 7% was introduced. After one second from the state in Fig. 2b, the strain in the NW surfaces was relaxed and the surface reconstruction on the lower side surface disappeared although the tensile strain was reduced but still remained on the top side surface, as observed in Fig. 2c. This implies that the magnitude of the stress applied to the top and bottom surfaces is different; a bending moment was introduced in the NW during tensile deformation. Because the NW length increased by 6.4 nm via 38 pulses, the average growth length resulting from a single pulse wave was calculated to be 0.17 nm, which is similar to the spacing of the (211) lattice plane. This implies that the NW length can be controlled by approximately monatomic layer spacing using the nanosecond-pulse wave.

**Figure 3:**
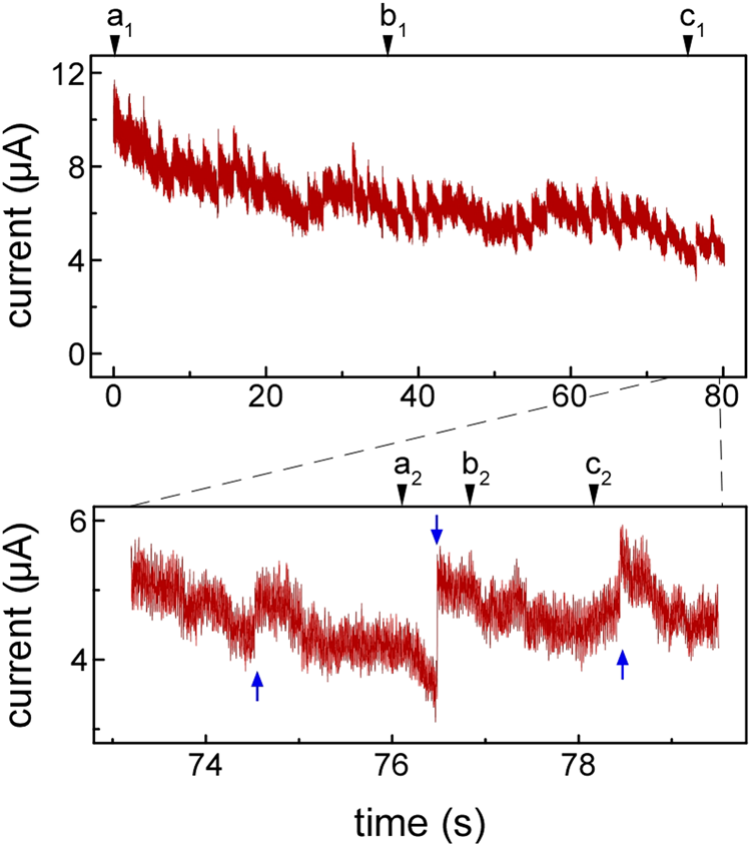
Variation in conductance during the NW formation shown in Fig. 1 (the lower frame corresponds to the process shown in Fig. 2). Times a_1_–c_1_ and a_2_–c_2_ indicate the observation times of Fig. 1a–c and Fig. 2a–c, respectively. The interval between each pulse is 2 s. A direct voltage of 1 mV is applied for conductance measurement. The arrows in the lower frame show the time and number of pulses. The conductance value increases rapidly by 0.5–1.5 μA at a 2 s interval pulse application, and subsequently decreases gradually until application of the next pulse voltage.

Figure 3 shows the variation in conductance during the NW formation shown in Fig. 1. In the lower frame of Fig. 3, the variation during the process shown in Fig. 2 is enlarged. Times a_1_–c_1_ and a_2_–c_2_ in Fig. 3 indicate the observation times of Fig. 1a–c and Fig. 2a–c, respectively. The interval between each pulse application is 2 s. Arrows in the lower frame of Fig. 3 show the time and number of pulses. The conductance value rapidly increases by 0.5–1.5 μA for each pulse, followed by a gradual decrease until application of the next pulse voltage.

The conductance of the NW rapidly increased during the pulse, as shown in Fig. 3. This is because the minimum cross-sectional width of the NW increased because of electromigration due to application of a pulsed voltage, as shown in Figs 1 and 2. Conversely, the conductance gradually decreased after the rapid increase in conductance, as the NW elastically thinned because of the tensile stress acting on the NW. Thus, the surface reconstruction shown in Fig. 2a,b occurred via the application of a pulsed voltage under tensile stress and disappeared during the structural relaxation process, as shown in Fig. 2b,c. Therefore, the observed surface reconstruction of the NW took place when electromigration occurred under tensile stress.

### The reconstruction structure in the NW

**Figure 4:**
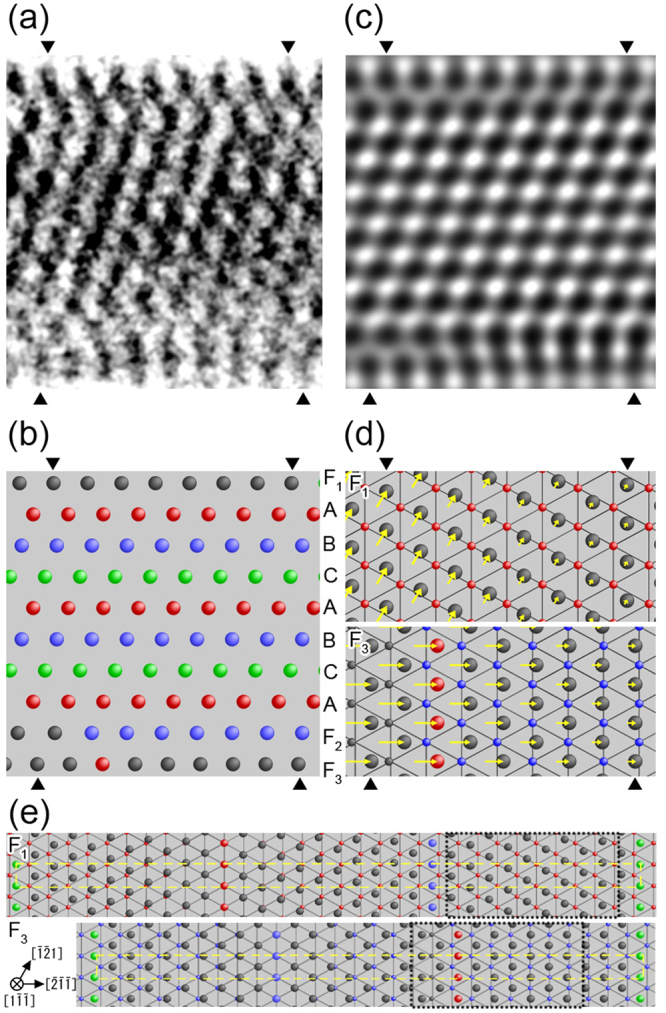
Analysis of the atomic configuration of the Au NW shown in Fig. 2b. (**a**) Enlarged high-resolution image of the region surrounded by the four triangles in Fig. 2b. (**b**) Atomic configuration model obtained from the high-resolution image in a. (**c**) Simulated image using the model in (**b**). The red, blue, and green circles represent the atom row positions of the three types of the ($1\bar{1}\bar{1}$) stacking layers in a fcc structure (A, B, and C). The black circles on the F_1_, F_2_, and F_3_ layers represent the atom row positions, which are different from those for the A, B, and C layers; F_1_, F_2_ and F_3_ layers are stacking faults. (**d**) Schematics representing the relationship between the atom positions of the F_1_ layer and the neighboring A layer (upper), and the relationship between the atom positions of the F_3_ and F_2_ layers (lower), viewed along the [$1\bar{1}\bar{1}$] direction. The larger circles correspond to the atoms on the F_1_ and F_3_ layers, respectively. The yellow arrows indicate the displacement from the ideal atom positions on the C layer. The displacement directions are the [$\bar{1}\bar{2}1$] and the [$\bar{2}\bar{1}\bar{1}$] directions for the F_1_ and F_3_ layers, respectively. (**e**) Virtually constructed atomic configuration models of the units of the surface periodic structures to explain the atomic configuration on the F_1_ layer (upper) and the F_3_ layer (lower). The models of the atomic configurations for the F_1_ and the F_3_ layers shown in (**d**) are included in the regions surrounded by the black dotted-line squares. The yellow broken-line squares represent the rectangular unit cells of the Au (111) surface reconstruction layers of the virtually constructed models. The unit cells are expressed as (${\rm{16}}\sqrt{3\,}\times \,1$) and (${\rm{14}}\sqrt{3}\,\times \,1$) for the models of the F_1_ and F_3_ layers, respectively.

Figure 4 shows analysis of the atomic configuration of the Au NW presented in Fig. 2b. We selected the core region of the wire, the part surrounded by four triangles in Fig. 2b, and show its enlarged high-resolution image in Fig. 4a. Because the thickness of the NW along the observed direction is several nanometers and the weak phase object approximation of the lattice imaging can be applied^18^, the positions of atom rows are identified from the black dots (the darkest) positions in the images. Thus, an atomic configuration model was obtained from the high-resolution image in Fig. 4a, as shown in Fig. 4b. Using this model, we simulated a high-resolution image at the Scherzer focus condition, as shown in Fig. 4c. The simulated image is well fitted to the observed image, implying that the model represents a suitable atomic configuration for the NW structure. In the model in Fig. 4b, the red, blue, and green circles represent the atom row positions of the three stacking layers of the ($1\bar{1}\bar{1}$) planes in a face-centered cubic (fcc) structure (the A, B and C stacking layers). Because the tensile stress was applied along the growth direction, i.e., the [211] direction, the (${\rm{211}}$) spacing of the interior layers, i.e., the spacing between the atom rows on the A, B and C stacking layers, expanded by 7% in comparison with that of the stress-free state, as shown in Fig. 2b. The atom row positions on the top and bottom side surface layers (the F_1_, F_2_, and F_3_ layers in Fig. 4b) are different from the atom row positions on the A, B, and C stacking layers; the F_1_, F_2_ and F_3_ layers are defined as stacking fault layers on the top and bottom side surfaces. The spacings between the atom rows on the F_1_ and F_3_ layers are 3% and 7% shorter than those on the A, B, and C stacking layers, respectively. Thus, the spacing on the F_1_ layer is 4% larger than that of the stress-free state, and that on the F_3_ layer is similar to that of the stress-free state. For the F_2_ layer, the atom row positions in the right side (the blue circles in Fig. 4b) are located at the atom row positions in the B stacking layer, i.e., most of the atom rows on the F_2_ layer are located at that of the B layer, whereas the atom row positions on the left side (the black circles in Fig. 4b) are displaced from the atom row positions in the B stacking layer. In Fig. 4d, we illustrate the relationship between the atomic positions of the top side surface layer (the F_1_ layer) and the neighboring A layer in the upper schematic, and the relationship between the atomic positions of the bottom side surface layer (the F_3_ layer) and the neighboring F_2_ layer in the lower schematic. These atomic positions are viewed along the [$1\bar{1}\bar{1}$] direction normal to the incident electron beam for TEM observation along the [$0\bar{1}1$] direction. The smaller circles on the triangular lattices in Fig. 4d correspond to the atoms on the A and F_2_ layers in the upper and lower schematics, respectively. The larger circles in Fig. 4d correspond to the atoms on the F_1_ and F_3_ layers in the upper and lower schematics, respectively. The arrows attached to the atoms indicate the displacement vectors from the atomic positions on the ideal surface, i.e., the C layer, which is a non-faulted fcc stacking layer close to the F_1_ and F_3_ layers. The atom row on the right side on the F_1_ layer approximately locates at the atom row position of the C stacking layer, whereas the atom row at the left side locates at around the atom row position on the B stacking layer. The B stacking position on the F_1_ layer corresponds to a hexagonal close-packed (hcp) stacking position. These atom rows on the F_1_ layer are displaced along the [$\bar{1}\bar{2}1$] direction so that the spacing between the atom rows along the [$0\bar{1}1$] direction in the middle region on the layer equilibrates, as shown by the yellow arrows attached to atoms in the upper schematic in Fig. 4d. The right end atom row on the F_3_ bottom layer is located at the middle position between the A and C stacking sites, whereas the third atom row from the left end on the F_3_ bottom layer is located at the A stacking site, corresponding to the hcp stacking site, and other atom rows on the F_3_ bottom layer are displaced along $[\bar{2}\bar{1}\bar{1}]$ so that each atom row spacing on the layer equilibrates, as shown by the arrows attached to atoms in the lower schematics in Fig. 4d. To analyze the atomic configuration on the F_1_ and F_3_ layers, we virtually constructed extended atomic configuration models of the units of the surface periodic structures, as shown in Fig. 4e. These models were constructed from the conditions obtained from the models shown in Fig. 4d: (1) the atomic spacing on the F_1_ and F_3_ surface reconstruction layers was determined to fit the observed results, and (2) both the right and left end atom rows were located at the C stacking position to superimpose the atomic positions of both end sides of the periodic structures, i.e., to define their unit cells. As a result, the models derived from the TEM observation shown in Fig. 4d are reproduced in the regions indicated by the black dotted-line squares in Fig. 4e. For the virtually extended models, the rectangular unit cells of the two Au {111} surface reconstruction layers are defined as indicated by the yellow broken-line squares in Fig. 4e. The unit cells are expressed as (${\rm{16}}\sqrt{3}\times 1$) and (${\rm{14}}\sqrt{3}\times 1$) for the upper and lower models in Fig. 4e, respectively. Because the side surface areas of the observed NW are smaller than those of the unit cells of the virtually extended models in Fig. 4e, the surface reconstruction observed in the NW cannot be defined using Wood’s notation. Instead, the observed surface reconstruction on the F_1_ and F_3_ layers can be expressed as portions of the unit cells of the virtually extended atomic configuration models.

## Discussion

The atom rows in the top and bottom surface layers of the observed Au NW are displaced from the atom row positions of the non-faulted fcc stacking layers. It is well-known that stacking faults are introduced in the interior of crystals to relieve stress caused by mechanical deformation and thermal treatment^19^. On relatively large surfaces, the displacement of atoms, i.e., surface reconstruction, occurs to stabilize surface structures. When the {111} surfaces in Au, which correspond to the reconstructed surface planes of the NW in this study, are sufficiently large, Au (111) – ($22\times \sqrt{3}$) type reconstruction occurs^20,21^. For this reconstruction, a compressive strain of approximately 4% is introduced to the surfaces, resulting in the formation of a huge unit cell consisting of fcc, hcp, and the bridge sites between them. The formation mechanism of the huge unit cell is interpreted based on long-range interactions, especially elastic stress domains^22,23^. In contrast, when the surfaces become smaller than the unit cells of such surface reconstructions, their stabilization is caused by continual motion of both surface and internal atoms, i.e., structural fluctuation, as observed in Au nanoclusters^24^. The driving force of the fluctuation is explained on the basis of the shear stress caused by Coulomb forces because of the charging up of the nanoclusters during electron beam irradiation^25^. The local heating by the electron beam has been thought to hardly affect to the fluctuation since the actual increase in the specimen temperature during the observation was less than ~370 K at usual irradiation density (~10^4^ A/m^2^)^26^. Thus, surface reconstruction and surface modification are caused by the relaxation of stress. In this study, surface reconstruction in the NWs was observed when pulsed voltages were applied under tensile stress; it is inferred that stress contributed to the surface reconstruction similar to other surface reconstruction and modulation observed for larger surfaces and nanoclusters. However, the Au(111) – ($22\times \sqrt{3}$) reconstruction observed on larger surfaces never forms in the NWs in this study because their width is at most 2–6 nm, considerably smaller than the unit cell of the Au(111) – ($22\times \sqrt{3}$) reconstruction, which has a domain width of ~15 nm. In addition, no Coulomb forces act on the present NWs because both ends of the NW were electrically connected to two Au nanotips (electrodes), which are semi-infinite conductors. Therefore, the surface reconstruction observed in this study is different from what has been previously observed.

The surface reconstruction observed in this study was caused by electromigration via pulsed voltage application under tensile stress conditions. Electromigration occurs predominantly at surfaces^17,27^. As observed in Fig. 2b, a bending moment was introduced in the NW, as the introduced strain on the surfaces is larger than that in the interior region of the NW. In particular, the expansion on the top surface was larger than that on the bottom surface. Such surface stresses can be relieved by rearrangement of the surface atom positions because the atoms move easily by electromigration. Therefore, it is inferred that the surface reconstruction observed in the NW surfaces is a peculiar phenomenon caused by the surface relaxation of tensile strain on Au surfaces via electromigration. The atomic displacement process can be described as follows. First, a tensile strain was introduced to the NW and the atoms on the monatomic surfaces moved by electromigration caused by application of pulsed voltage to reduce the tensile stress. Because of the strain relaxation, the surface atoms of the NW were displaced from the fcc stacking sites of the {111} planes, followed by the formation of long bridge sites; then surface reconstruction occurred. After the subsequent displacement of the surface atoms, the strain of the bottom surface was relieved perfectly and that on the top surface was reduced by half. Although the strain on the top surface remained, it was smaller than that in the interior region. Thus, the top surface layer was also relaxed in this state.

As shown in Fig. 4e, the observed surface reconstruction on the F_1_ and F_3_ layers can be expressed as portions of the unit cells of the virtually extended atomic configuration models. Although the size of the unit cells of the virtual models, i.e., Au (111) – (${\rm{14}}\sqrt{3}\times 1$) and Au (111) – (${\rm{16}}\sqrt{3}\times 1$), are similar to that of the Au (111) – ($22\times \sqrt{3}$) unit cell observed on sufficiently large Au (111) surfaces^20,21^, the reconstruction unit cells are different. This implies that when the Au (111) surface areas become smaller than the area of the unit cells of sufficiently large Au (111) surfaces, the surface reconstruction manner changes. It can be deduced that when the side surface areas of NWs become sufficiently large, the Au (111) – ($22\times \sqrt{3}$) type surface reconstruction appears. In contrast, when NWs become smaller than those observed in this study, their surface structures destabilize the interior of the NWs, leading to the onset of structural fluctuation throughout the entire regions^24^. In such states, surface reconstruction cannot be defined. Thus, the surface reconstruction of the NW observed in the present study can emerge only when the NW surfaces satisfy a critical size condition such that surface and bulk can be distinguished.

## Summary

In this study, the atomic behavior during the growth of Au NWs via the application of pulsed voltages under tensile stress was observed *in situ* by transmission electron microscopy. A Au NW of 6.4 nm in length and 2.3 nm in width was formed after 38 pulses. Using this pulsing method, the average growth speed was controlled with an accuracy on the order of the atomic plane spacing, i.e., spacing of the (211) plane (0.17 nm). We found that the surface monatomic layers were reconstructed by stress application and subsequent relaxation via electromigration. Such atomic control of the shapes and surface structures of NWs can be directly applied to assembling NWs for electrical circuits and to their stress relaxation. Also, the unprecedented surface reconstruction structures of Au NWs lead to new mechanical, catalytic, and light emission properties^28–31^. The results of this study, revealing unprecedented reconstruction of NW surfaces, inspire the study and development of new NW structures and corresponding useful functions for nanodevices.

## Methods

### Experiment

We used the high-resolution TEM at the University of Tsukuba, which is equipped with dual goniometer stages that enable picometer-precise manipulation of two nanotips^9,19,32,33^. Rectangular pulse waves were applied to Au NCs while tension was applied by pulling back the maicrocantilever tip to produce NWs. We observed the formation process of NWs *in situ* by lattice imaging (see Fig. 1S in Supplementary Information).

### Image simulation

Simulation of high-resolution TEM images was carried out using xHREM^TM^ (HREM Research Inc., version 3.5.2.5)^34^.

## Electronic supplementary material

Supplementary Information

Movie 1

Movie 2
