# Supplementary material for: Surface reconstruction in gold nanowires
Source: Sci Rep. 2018 Jun 29;8:9836. doi: 10.1038/s41598-018-28145-y (PMC6026130; doi:10.1038/s41598-018-28145-y)
Supplement: Supplementary file 1 — Supplementary Information [file 41598_2018_28145_MOESM1_ESM.pdf]

Supplementary Information

Title: Surface reconstruction in gold nanowires

Authors: Yasuchika Suzuki and Tokushi Kizuka

The Supplementary Information includes the title and captions of Movies 1 and 2 corresponding to Figs. 1 and 2 and the figure of experimental setup and its caption, as follows.

Movie titles and captions

Movie 1 **Movie of *in situ* high-resolution TEM of the formation of a Au NW during the application of pulsed voltage under tensile stress, corresponding to Fig. 1.**

The pulse trigger is displayed in the lower window. The width and height of the applied pulse waves are 4 ns and 0.60 V, respectively. The replay speed is real time.

Movie 2 **Movie of *in situ* high-resolution TEM of the surface reconstruction and subsequent relaxation of the Au NW during one pulse under tensile stress, corresponding to Fig. 2.**

The pulse trigger is displayed in the lower window. The width and height of the applied pulse waves are 4 ns and 0.60 V, respectively. The replay speed is a third of real time.

Fig. S1 and its caption

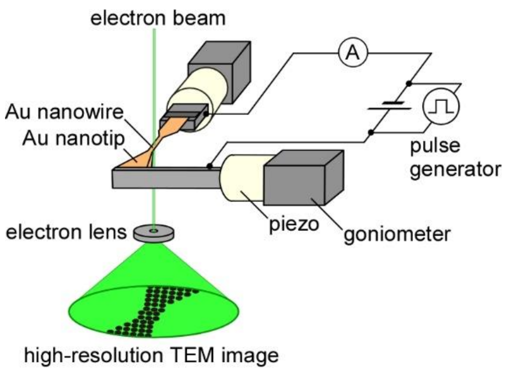

Fig. S1 The setup of the experiment. Two different types of Au nanotips were prepared: a Au-covered silicon nanotip attached to a silicon cantilever for atomic force microscopy, and a Au plate, the edge of which was thinned to several nanometers using argon ion milling to produce a nanotip. These nanotips were first inserted into a TEM specimen chamber and a NC was established by contacting each tip using goniometer and piezo manipulation. Then, rectangular pulse waves were applied to the NC while tension was applied by pulling back the cantilever tip to produce NWs. We observed the formation process of NWs *in situ* by lattice imaging. The conductance of the NWs was measured by applying a potential of 1 mV. The temperature and pressure in the specimen chamber were room temperature and 10^{-5} Pa, respectively.
